# Harmonization of monographic standards is needed to ensure the quality of Chinese medicinal materials

**DOI:** 10.1186/1749-8546-4-18

**Published:** 2009-09-22

**Authors:** Kelvin Chan, Kelvin Sze-Yin Leung, Sandy Shuo Zhao

**Affiliations:** 1 Department of Pharmacy, School of Applied Sciences, University of Wolverhampton, Wolverhampton WV1 1LY, UK; 2 Department of Chemistry, Hong Kong Baptist University, Kowloon Tong, Kowloon, Hong Kong, PR China

## Abstract

This article provides an overview on the regulations of Chinese medicinal materials (CMMs) in various countries and regions. Harmonization of CMM monographs would provide standards for the quality control of CMM products and play an important role in the modernization and globalization of Chinese medicine. A harmonized regulatory system would improve the quality of CMMs thereby ensuring the safety of the products and assisting Chinese medicine practitioners in their practice. The fast growing demand worldwide for traditional medicines calls for harmonized monographic standards to safeguard the safety and quality of CMM products.

## Background

Seventy to eighty percent of the world population relies on non-conventional medicines (mainly of herbal sources) as their primary health care [1]. Chinese medicinal materials (CMMs) have the highest turnover-trading-figure among all herbal medicines [1].

At present there is a lack of methodology for the quality control of CMMs. Most pharmacopoeias merely state the minimum requirements to safeguard public safety. To prevent adulterated CMMs, manufacturers must adopt adequate quality control of international standards for harvesting, collecting, processing and packing of the crude herbs and final products. Licensing and registration of herbal medicine are required to enforce the quality assessment of CMMs. Specific monographic profiles of CMMs can standardize the authentication and quality assessment for CMM manufacturers worldwide.

### Herbal standards around the world

Differences among national or regional regulations on import and export of medicinal plants can affect the quality control of herbal products. Same medicinal plant products may be classified as food, food-supplements, functional food, nutriceuticals or prescription herbal medicines in different countries or regions. The key features of such diversified national and regional practices due to different monographic standards on CMM products are summarized in Table 1.

**Table 1:** Pharmacopoeia or standards of various countries or regions that have monographic standards for CMMs

**Pharmacopoeia and monograph**	**Authority**	**Status**
*WHO Monographs on Selected Medicinal Plants*	World Health Organization	Unofficial
*Chinese Pharmacopoeia*	State Food and Drugs Administration, China	Official
*Australian Regulatory Guidelines for Complementary Medicines*	Therapeutic Goods Administration, Australia	Official
*European Pharmacopoeia*	European Directorate for Quality Medicines and Healthcare	Official
*Hong Kong Chinese Materia Medica Standards*	Department of Health, Hong Kong, China	Official
*Japanese Pharmacopoeia*	Pharmaceutical Affairs, Japan	Official
*Thai Herbal Pharmacopoeia*	Thai Food and Drug Administration, Thailand	Official
*British Pharmacopoeia*	British Pharmacopoeia Commission, UK	Official
*American Herbal Pharmacopoeia (AHP)*	USA	Unofficial

#### World Health Organization

Over the years, the World Health Organization (WHO) has introduced monographs of medicinal plants used around the world. WHO also maintains a list of herbs that are widely used in primary health care in various countries as a result from the *WHO Guidelines for the Assessment of Herbal Medicines *which promotes the development of monographs to standardize the quality control of herbal medicines. Twenty-five monographs encompassing 28 plants have been published in Volume I [2] and monographs of 30 plants have been included in Volume II [3]. WHO emphasizes that these publications are not 'intended to replace official compendia such as pharmacopoeias, formularies, or legislative documents but to promote harmonization in the use of herbal medicines with respect to levels of safety, efficacy, and quality control' [3].

#### Mainland China

China's first drug control law was promulgated in 1984 [4]. According to the law, production of a new drug is subject to approval by the Drug Regulatory Department under the State Council. The drug regulatory department has compiled a list of crude Chinese medicines. A manufacturer may start producing a drug after a registered number is granted. If a manufacturer modifies the production process, approval from the authorities is necessary. *Pharmacopoeia of the People's Republic of China *[5] is compiled by the Drug Regulatory Department according to the national drug standards in China [6]. The Drug Administration Law of the People's Republic of China was implemented in 2002 [7].

Good Agricultural Practice (GAP) is also applicable to the quality control of Chinese crude drugs [8] as it includes quality aspects such as macroscopic/microscopic authentication, chemical identification, bioactive compounds and metal elements, as well as pesticide detection. Microscopic examination/authentication identifies the characteristics of tissues, cells or cell contents in sections, powders or surface on slides of CMMs. Chemical identification should include high performance liquid chromatography (HPLC) fingerprints and Fourier transform infrared spectroscopy (FTIR) in investigation stage [9]. Bioactive compounds should be assayed [10]. In China, the authorities have implemented GAP for the cultivation of over 80 species of commonly used CMMs in regions where CMM plants are traditionally cultivated. Outside China CMM plants are cultivated to meet the increasing demand; however, no consensus in methodology has been reached as to how effective regulation can reflect the multi-bioactivity aspects of CMMs [11].

#### Australia

Therapeutic Goods Administration (TGA) under the Commonwealth Department of Health is the national therapeutic goods control authority in Australia. The *Australian Regulatory Guidelines for Complementary Medicines *(ARGCM) [12] is used to regulate Chinese medicine which is classified as a complementary medicine [13]. The regulatory framework for complementary medicines in Australia is a two-tier one, classifying registered medicines into high risk or low risk groups [14]. Risk assessment is conducted on ingredients, indications and claims, dosage form, significance of side effects and effects of prolonged use or from inappropriate self-medication. Therapeutic Goods Act 1989 requires that therapeutic goods available in Australia should be included in the Australian Register of Therapeutic Goods (ARTG), unless they are specifically exempted from this requirement by Schedule 5 of the Therapeutic Goods Regulations 1990 [13].

#### Continental Europe

In 1964, Belgium, France, Germany, Italy, Luxembourg, the Netherlands, Switzerland and the United Kingdom signed the Convention on the Elaboration of a *European Pharmacopoeia *which has been ratified in 31 European states [15]. *European Pharmacopoeia *is now a standard reference for both European and non-European countries [16] and published by the European Directorate for the Quality of Medicines and Healthcare (EDQM) [17]. *European Pharmacopoeia *is known for its universal requirement of all medicines regardless of their origins [15]. About 130 herbal medicines including drugs and drug preparations are included in the *European Pharmacopoeia *[15]. All necessary tests and assay methods described in the monograph were rigorously validated according to the *Technical Guide *[18]. In response to toxicity incidences of herbal products, the European Commission requested that the monographs on herbal drugs used in traditional Chinese medicine should be developed to achieve a modern quality standard according to the *European Pharmacopoeia*, and listed the herbal drugs subject to investigation [19]. Currently the emphasis has been placed on compiling a list of all herbal drugs subject to investigation. The European Pharmacopoeia Commission is producing more and more monographs and elaborating monographs on proprietary drugs [19].

#### Hong Kong, China

In 1999, the statutory status was accorded to Chinese medicine in Hong Kong and the Chinese Medicine Council of Hong Kong (CMC) was established [20] to regulate Chinese herbal medicines with assistance from the Chinese Medicine Division of the Department of Health [21]. All proprietary Chinese medicines (PCMs) manufactured or sold in Hong Kong must be registered. For PCMs registered under 'new drug category', additional supporting information such as acute/chronic toxicities should also be provided [20].

In 2001, the Department of Health published the *Hong Kong Chinese Materia Medica Standards *(HKCMMS) [22] which is currently the only standard that has comprehensive limits for heavy metals, pesticide residues and mycotoxins across all monographs (Table 2). All analytical methods and parameters under the HKCMMS were advised by the International Advisory Board (IAB) after considering all the data generated by research efforts from experts of the six universities in Hong Kong. In addition, a Scientific Committee, consisting of IAB members and representatives of the participating universities and government departments, were set up to resolve technical issues and examine research results. All limits were determined with ten samples and with reference to the *Chinese Pharmacopoeia, British Pharmacopoeia*, *European Pharmacopoeia*, *Japanese Pharmacopoeia *and *US Pharmacopoeia *[22] (Table 2).

**Table 2:** Hong Kong Chinese Materia Medica Standards recommended limits of heavy metals, pesticide residues and mycotoxins

**Heavy metal**	**Limits**
Arsenic	2.0 mg/kg
Cadmium	0.3 mg/kg
Lead	5.0 mg/kg
Mercury	0.2 mg/kg
	
**Pesticide**	
Aldrin and Dieldrin (sum of)	0.05 mg/kg
Chlordane (sum of *cis- trans*- and oxychlordane)	0.05 mg/kg
DDT (sum of p,p"-DDT, o,p'-DDT, p,p'-DDE and p,p'-TDE	1.0 mg/kg
Endrin	0.05 mg/kg
Heptachlor (sum of heptachlor and heptachlor epoxide)	0.05 mg/kg
Hexachlorobenzene	0.1 mg/kg
Hexachlorocyclohexane isomers (α-, β- and δ-hexachlorocyclohexane)	0.3 mg/kg
Lindane (γ-Hexachlorocyclohexane)	0.6 mg/kg
Quintozene (sum of quintozene, pentachloroaniline and methyl pentachlorophenyl sulphide)	1.0 mg/kg
	
**Mycotoxin**	
Aflatoxin B_1_	5 μg/kg
Aflatoxins (sum of B_1_, B_2_, G_1 _and G_2_)	10 μg/kg

#### Japan

In Japan, *Kampo *medicine refers to Chinese medicine and Japanese indigenous medicine [23]. *Kampo *formulae had been non-prescription medicines until 1985 when certain *Kampo *medicines became classified as prescription medicines and are therefore subject to clinical evaluation [24]. A total of 148 *Kampo *formulae have been approved for clinical use in Japan. The monographs of the top 20 *Kampo *extracts have been published in the latest version of the *Japanese Pharmacopoeia *as the official standards for the medically significant herbal substances [25].

#### Thailand

In Thailand, medicinal plant materials or crude drugs used in traditional medicines are exempt from registration for easy public use [26]. Prior to the production of any traditional medicine, manufacturers must apply for the manufacturing licenses from the Thai Food and Drug Administration [27]. The registration requires information on the raw material or ingredients, method of process, dosage and quality control. Furthermore, safety information related to acute, sub-chronic and chronic toxicity test as well as clinical trials results should be provided [28]. *Thai Herbal Pharmacopoeia *is published by the subcommittee on the establishment of the *Thai Herbal Pharmacopoeia *under the supervision of the Thai Pharmacopoeia Committee [29]. *Thai Herbal Pharmacopoeia *covers 23 monographs of Thai medicinal plant materials and three herbal preparations. Currently, compliance with the GMP and other standards for the manufacture of traditional medicines are voluntary; however, traditional medicines submitted for registration must pass the limited tests of microbiology, heavy metal and pesticide residues. In addition, the Thai Food and Drug Administration is a member of the Uppsala Monitoring Center Network responsible for the surveillance of the safety of health care products [29].

#### United Kingdom

In the United Kingdom, there are three regulatory routes for herbal medicines: unlicensed herbal remedies, registered Traditional Herbal Medicines (THMs) and licensed herbal medicines [30]. Herbal products do not have to meet specific standards of safety and quality. Registered THMs are regulated by the Traditional Herbal Medicines Registration Scheme and are required to meet specific safety and quality standards and to be accompanied by agreed indications. Licensed herbal medicine must have a product license or marketing authorization. *British Pharmacopoeia *contains approximately 3100 monographs whereby all medicines and health care products are regulated [31]. CMMs are classified under the herbal and complementary medicines division in the *British Pharmacopoeia*.

At present, the *British Pharmacopoeia *contains 13 monographs of traditional herbal medicines which facilitate assessment of registration applications and gives a reference standard to inform the manufacturers and importers of the UK regulations. For the first time, a monograph of *Radix et Rhizoma Glycyrrhizae *(*Gancao*, Liquorice root), a Chinese medicinal herb, was introduced into the *British Pharmacopoeia*. Furthermore, collaboration has been established between the British and Chinese Pharmacopoeias in order to exchange information on quality standards for medicines, develop test methods, identify common adulterants or impurities and to authenticate herbal materials [32].

#### United States

In the United Sates, CMMs are classified as supplementary products regulated by the Dietary Supplements Health and Education Act (DSHEA) [33]. A manufacturer must guarantee that the product is safe and properly labeled. While approval from the Food and Drug Administration is not required, new dietary ingredients are required for pre-market safety review [33]. Overall, the regulation for dietary supplements is less stringent than that for drugs.

In the United Sates, the *American Herbal Pharmacopoeia *(AHP) publishes monographs for herbs used as dietary supplements. AHP offers standard herbal monographs whereby a genus and species may be identified according to the Lingnean system of botanical classification and nomenclatures [34]. AHP also produces monographs on herbs and other botanical ingredients, not necessarily already in the AHP [35]. AHP has published several monographs on botanicals in its dietary supplement section [34].

## Discussion

The present article provides an overview on the regulations of CMMs in various countries and regions. Each individual regulation system focuses on specific issues. In the United Sates, regulation places its emphasis on source herbal materials. In the European Union, procedures focus on authentication of herbal materials. The European Medicine Evaluation Agency comprising EU member states was formed for managing the *European Pharmacopoeia*. A *Technical Guide *was issued with all technical details on the scientific works developed for those medicinal materials under regulations. In Australia, TGA regulates all the registered products in terms of the quality, safety and efficacy. In the UK, regulation focuses on safety evaluation. In China, the regulation is directed to proper formulation of CMM products according to traditional Chinese medicine theory. Under the present systems herbal manufacturers can submit their products according to the ease of getting registration in the regions where they can market or sell their products. One of the Chinese medicine practices is composite herbal formulae (*Fufang*) for individualized treatment. If the quality of CMMs is not standardized, treatment variability will exist in addition to other variables. It is imperative, therefore, for regulatory agencies worldwide to set up harmonized regulatory controls over the manufacture and trade of CMMs.

## Conclusion

The fast growing demand worldwide for traditional medicines calls for harmonized monographic standards to safeguard the safety and quality of CMM products.

## Competing interests

The authors declare that they have no competing interests.

## Authors' contributions

All authors took part in the discussion before drafting the present article. KSYL and SSZ did literature review on national standards. KC provided information on current aspects in various sections. All authors read and approved the final version of the manuscript.
